# Physical Activity in People with Multiple Myeloma: Associated Factors and Exercise Program Preferences

**DOI:** 10.3390/jcm9103277

**Published:** 2020-10-13

**Authors:** Jennifer L. Nicol, Carmel Woodrow, Nicola W. Burton, Peter Mollee, Andrew J. Nicol, Michelle M. Hill, Tina L. Skinner

**Affiliations:** 1 School of Human Movement and Nutrition Sciences, The University of Queensland, Brisbane 4072, Australia; anic9909@bigpond.net.au (A.J.N.); t.skinner@uq.edu.au (T.L.S.); 2 Department of Haematology, Division of Cancer, Princess Alexandra Hospital, Brisbane 4102, Australia; Carmel.Woodrow@health.qld.gov.au (C.W.); Peter.Mollee@health.qld.gov.au (P.M.); 3 School of Applied Psychology, Griffith University, Brisbane 4111, Australia; n.burton@griffith.edu.au; 4 Faculty of Medicine, The University of Queensland, Brisbane 4006, Australia; 5 Brisbane Clinic for Lymphoma, Myeloma and Leukaemia, Greenslopes Private Hospital, Brisbane 4120, Australia; 6 Precision and Systems Biomedicine Laboratory, QIMR Berghofer Medical Research Institute, Brisbane 4006, Australia; Michelle.Hill@qimrberghofer.edu.au

**Keywords:** multiple myeloma, exercise, symptoms, supportive care, quality of life, well-being

## Abstract

People with multiple myeloma (MM) often experience disease symptoms and treatment toxicities that can be alleviated through physical activity (PA). However, the majority of people with MM are insufficiently active. This study explored PA among people with MM, including differences by treatment stage, symptoms and demographics, and programming preferences. Overall, 126 people with MM (77% response rate) completed the survey. Pre-diagnosis, 25.4% were sufficiently active, with 12.0% remaining active after treatment. Respondents who were physically active pre-diagnosis were 46.7 times (95% confidence intervals CI: 2.03, 1072.1) more likely to meet PA guidelines following an MM diagnosis compared to people not meeting guidelines pre-diagnosis. Experiencing MM symptoms and receiving PA advice from healthcare professionals were not associated with meeting PA guidelines. People with MM were interested in exercise programs (55%) that are low-cost (77%), offered at flexible times (74%), and at locations close to home (69%), both during active treatment and remission (57%), and supervised by an exercise oncology specialist (48%). People with MM, particularly those insufficiently active prior to diagnosis, should be offered convenient, low-cost exercise programs supervised by an exercise oncology specialist to increase PA participation.

## 1. Introduction

Multiple myeloma (MM), an incurable cancer of plasma cells, is a disease characterised by a progressive sequence of remission and relapse, with constantly changing treatment schedules and a continually worsening prognosis. People with MM often experience severe effects of the disease that adversely affect their quality of life [1]. For example, a common adverse effect of MM is lytic lesions in the long bones that are associated with spine, rib and hip fractures, anaemia, low platelets, and renal failure [2]. Furthermore, the wide variety of treatments available for people with MM produces significant side effects and toxicities that are detrimental to quality of life [3]. Indeed, a comparison of the quality of life profiles from older survivors across 12 cancer types indicated that people with MM reported the greatest physical and mental health impairments [4].

Previous research in people with MM [5,6,7] has shown that physical activity (PA) is associated with improved quality of life outcomes. A recent population-based study of people with MM reported that those who do regular PA report better global quality of life and a reduced burden of both fatigue and disease-specific side effects from previous treatments [8]. However, only one in five people with MM have previously been reported to meet PA guidelines of at least 150 min/week of moderate or higher intensity PA during off-treatment periods [6].

Given these clear benefits of PA for people with MM, it is important to identify and subsequently address the modifiable factors associated with PA participation in this population. Previous research in this area has been limited and conflicting. A 2004 survey of 88 Canadian MM survivors (response rate = 56.4%) found no association between any demographic or medical characteristic and PA [6]. In a 2011 Australian study (*n* = 229, response rate not reported) [9], meeting the PA guidelines prior to diagnosis was the only variable that predicted PA participation post-diagnosis. However, neither of these studies investigated the impact of MM symptoms and treatment-related side effects on post-diagnosis PA, with both conducted prior to remarkable advancements in therapies that have vastly improved MM survival rate. The impact of these new treatment options and subsequent new treatment-related side effects on PA participation has yet to be elucidated. Furthermore, whether these treatment-related side effects and other relevant factors influence exercise program interest and uptake for people with MM requires further exploration.

The aim of this study was to explore PA among people with MM, including differences by treatment stage, MM symptoms, associated demographic factors and PA advice from healthcare professionals. Interest in, and preferences for, exercise programs were also determined.

## 2. Experimental Section

### 2.1. Participants

Men and women aged 18 years and older with a diagnosis of MM were invited to participate in the study. People were ineligible if they were unable to understand the English language or were not cognitively capable of providing consent. People with MM were identified by the clinic nurses during routine visits to Myeloma Clinics at public and private hospitals, and through community seminars conducted by the Leukaemia Foundation of Australia and Myeloma Australia in Brisbane, Australia. Details of the aims and requirements of the research were provided in the Participant Information Sheet at the beginning of the survey. Formal consent was obtained prior to completing the survey. The protocol was approved by the Greenslopes Private Hospital (#17/62), Metro South Research Ethics Committee (#HREC/18/QPAH/345) and the Human Ethics Committee of The University of Queensland (#2018000883/17/62). The study was conducted in accordance with the principles of the Declaration of Helsinki of 1975, revised in 2013.

### 2.2. Study Instrument and Outcomes

Data were collected using a self-administered paper version of the survey (32 items) (Supplementary Data: Tables S1–S5). The survey included four sections:

Section 1 (Items 1–8)—Participant demographics (age, gender), current living situation (household members, area of residence, type of dwelling), highest level of education, and mode of transport to medical consultations.

Section 2 (Items 9–13)—Self-reported health- and disease-related characteristics: treatment stage, time since diagnosis, current and previous MM treatment, and intensity of each of 13 symptoms related to MM experienced in the past month (classified as “not at all”, “slightly”, “moderately”, “severely”, and “overwhelmingly”). Myeloma treatment stages were classified as “smouldering”, “newly-diagnosed”, “non-active”, or “relapsed” disease and were determined from whether the respondent was undergoing active treatment for myeloma (item 9) and what treatment they had previously received (item 12). Smouldering included people with blood and bone marrow parameters indicative of a diagnosis of MM and who do not yet fulfil the criteria for treatment. Newly-diagnosed included people with MM who were receiving their first course of treatment and had never been in remission. Non-active included people with MM who were not currently on treatment. Relapsed included people with MM on treatment within their second and subsequent stages of therapies. The presence of each MM symptom was dichotomised to 1 (“moderately”/“severely”/“overwhelmingly”) and 0 (“not at all”/“slightly”). In addition, a categorical variable (“yes”/“no”) for each respondent was created for the presence of any MM symptom experienced to at least a moderate degree.

Section 3 (Items 14–19)—Participants were asked to describe frequency and duration of PA carried out for each of before MM diagnosis, during active treatment, and after treatment completion. Light, moderate (e.g., fast walking, tennis, and easy bicycling), and vigorous (e.g., running, jogging, and vigorous swimming) PA in a typical week was self-reported using the Godin Leisure Time Physical Activity Questionnaire (Godin LTPAQ) [10]. The Godin LTPAQ has been shown to have a modest correlation with accelerometry-derived measures of PA of at least moderate intensity in colon cancer survivors (rho = 0.51) [11] and has been used to measure PA in people with myeloma [6,9]. Meeting PA guidelines was defined as the accumulation of 150 min/week of at least moderate intensity PA, which was derived for before and after diagnosis and during periods off-treatment by summing time spent in moderate and vigorous activity, with minutes in vigorous activity weighted by two to account for greater intensity [12].

Section 4 (Items 20–32)—Interest, advice on PA participation received from health professionals since diagnosis of MM, and preferences. Responses were collected using a 5-point Likert scale. The survey items aligned with previous literature on the attitudes and preferences of people with MM regarding exercise and PA participation [9,13].

#### 2.2.1. Interest (1 Item)

A single item measured the interest in attending an exercise program for people with MM, of which responses ranged from 1 (strongly disagree) to 5 (strongly agree). Due to the negative wording of the question, reverse scoring was used, with the proportion interested determined from those who responded ”strongly disagree” and ”disagree”.

#### 2.2.2. Frequency of Receiving Advice and Referral (2 Items)

Frequency of receiving PA and exercise advice from healthcare professionals and recommendations to see an exercise specialist (e.g., exercise physiologist/physiotherapist). Respondents were asked to consider whether they had received PA and exercise advice since their diagnosis of MM from a health care professional—e.g., myeloma specialist, rehabilitation specialist, general practitioner, physiotherapist, exercise physiologist, nurse. A single response was collected (”yes”, ”no”, ”unsure”), which was dichotomised for the logistic regression analysis to ”yes” and ”no/unsure”. For the frequency of referral, respondents were asked to consider how often they had been recommended by their myeloma specialist to see an exercise specialist (e.g., exercise physiologist or physiotherapist) for PA and exercise advice, with responses ranging from 1(”never”), 2 (”rarely”), 3 (”occasionally”), and 4 (”always”) to 5 (”only when I asked”). For the purposes of the regression analysis, a dichotomous variable (”yes”/”no”) was generated with recommendations considered to have been received for those who responded ”occasionally”, ”always”, or ”only when I asked”.

#### 2.2.3. Preferences (24 Items)

Preferences regarding exercise programs for people with MM—e.g., timing with respect to disease stage, location, and context. For location, timing, and aspects of program delivery, responses ranged from 1 (strongly disagree) to 5 (strongly agree). For features of the exercise program which were important to respondents for participation (e.g., cost, flexibility), responses ranged from 1 (very unimportant) to 5 (very important). For determination of proportions, categories ”strongly agree”/”agree” and ”very important”/”important” were collapsed into ”agree” and ”important”.

### 2.3. Statistical Analyses

Statistical analyses were performed with the Stata statistical software package, Version 15.0 (StataCorp., College Station, TX, USA). The characteristics of the study sample, including treatment stage and symptom intensity, were analysed using descriptive statistics. For data presentation and interpretation, proportions were calculated. Representativeness of the study sample was assessed by comparing data on variables age and gender with data from a nationwide audit of cancer in Australia [14]. Survey responses were collapsed into three categories: agree/confident (responses 4–5), neutral/unsure (3), and disagree/unconfident (1–2) for Likert scale items. Symptom intensity was described as ”not at all” (1)/”slightly” (2)/”moderately” (3)/”significantly” (4)/”overwhelmingly” (5) and collapsed into three categories: not at all (response 1), slightly (response 2), and moderately or greater (responses 3, 4, and 5). Time in years since diagnosis was presented as the median and range.

Where data were not normally distributed, nonparametric tests, including the Wilcox signed rank test were used to examine differences between pre-diagnosis and current participation in PA (total minutes and percentage completing sufficient PA). Interest in participating in an exercise program and treatment stage was assessed using tests of proportion and paired t-tests as appropriate.

A logistic regression analysis was conducted to assess what factors were associated with being sufficiently active during active treatment. Due to insufficient numbers, a logistic regression analysis was not conducted to investigate factors that were associated with being sufficiently active during off-treatment periods. Potential predictors (independent variables) included age, gender, living arrangement, time since diagnosis, treatment stage, highest level of education, presence of MM symptoms, receiving PA advice and/or referral to exercise professionals from health professionals, and whether the person met PA guidelines prior to their diagnosis. The dependent variable was current level of physical activity and was dichotomised into meeting versus not meeting PA guidelines. Respondents with a disease stage of ”smouldering” were not included in the regression analysis as they had not yet commenced active treatment. Adjusted odds ratios are presented with 95% confidence intervals (95% CI). All statistical tests were two-tailed with an alpha level of 0.05 set for statistical significance.

## 3. Results

### 3.1. Sample Characteristics

The demographics of survey respondents are described in Table 1 (response rate = 77%; invited: *n* = 163; consented: *n* = 143; provided data: *n* = 126). The majority of participants were men (65.9%) aged 64.3 (SD 10.3) years, which is not significantly different (*p* = 0.20) from the Australia-wide proportion of men with MM (56.4%) [14]. The median time since MM diagnosis was 3.3 years (range 0.1 to 21.8 years).

### 3.2. Physical Activity Participation

**Figure 1:**
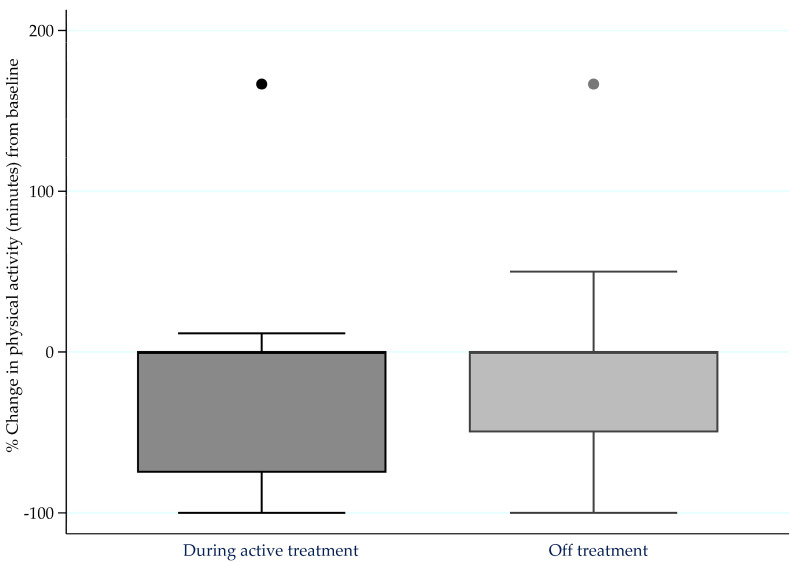
Percentage change in moderate-to-vigorous intensity physical activity (minutes) from pre-diagnosis levels to during active treatment (*n* = 109) and whilst off treatment (*n* = 70) for more than four weeks.

The proportions of people with MM who were meeting PA guidelines [12] before diagnosis, and during and after treatment, are presented in Figure 1. Before diagnosis, 26.5% were sufficiently active, which reduced to 12.7% after diagnosis during treatment (*p* < 0.001). After diagnosis, there was a slightly higher proportion of respondents who were sufficiently active off treatment (16.9%) compared to on treatment, but still significantly lower than the pre-diagnosis PA levels (*p* < 0.001).

### 3.3. Symptoms

**Figure 2:**
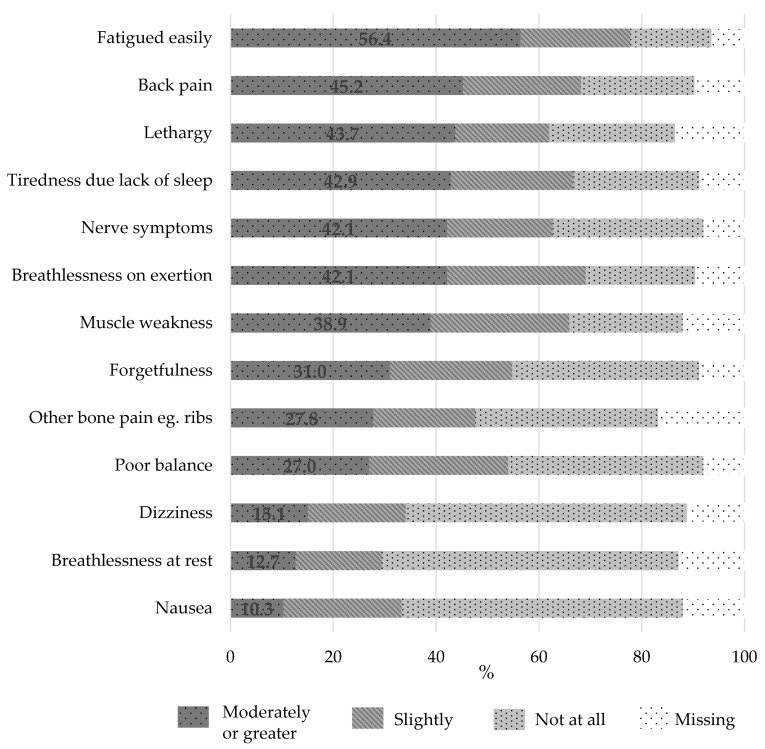
Intensity of symptoms experienced by people with multiple myeloma over the previous month (*n* = 126).

Intensity of MM symptoms experienced by the participants in the past month is presented in Figure 2. The majority of respondents experienced at least one symptom to a moderate intensity (84.5%), with 100% of respondents experiencing at least one symptom to a slight degree. Fatigue of at least moderate intensity was reported by 56.4% of respondents. Back pain (45.2%), lethargy (43.7%), and tiredness due to lack of sleep (42.9%) of at least moderate intensity were also commonly reported symptoms.

Less than half (42.1%) of the respondents reported receiving PA or exercise advice from any healthcare professional (e.g., myeloma specialist, rehabilitation specialist, general practitioner, physiotherapist, exercise physiologist, nurse) since their diagnosis. Only 14% of respondents had been referred to an exercise professional (exercise physiologist or physiotherapist), with another 4.8% referred only when they requested a referral.

### 3.4. Predictors of Physical Activity Participation

Using regression analysis, 57.2% (pseudo R squared) of the variance in PA participation was explained by the factors described in Table 2 (χ^2^ [15, *n* = 88] = 40.13, *p* < 0.001). The only significant predictor of PA after diagnosis was pre-diagnosis PA level. Controlling for all other variables in the model, people with MM who participated in sufficient levels of physical activity prior to their diagnosis were 46.7 (95% CI: 2.03, 1072.1) times more likely to currently meet physical activity guidelines compared to people who did not meet guidelines prior to diagnosis. No other individual variable explained whether respondents met PA guidelines, including whether or not they discussed PA with a healthcare professional or were experiencing MM symptoms to a moderate or greater intensity (*p* > 0.05).

When the influence of individual symptoms was assessed, the presence of fatigue (*p* = 0.014), back pain (*p* = 0.03), and other bone pain (*p* = 0.04) to at least a moderate degree was significantly associated with reduced odds of meeting PA guidelines during active treatment after diagnosis. However, after Bonferroni correction, no associations remained significant.

### 3.5. Exercise Program Preferences

**Figure 3:**
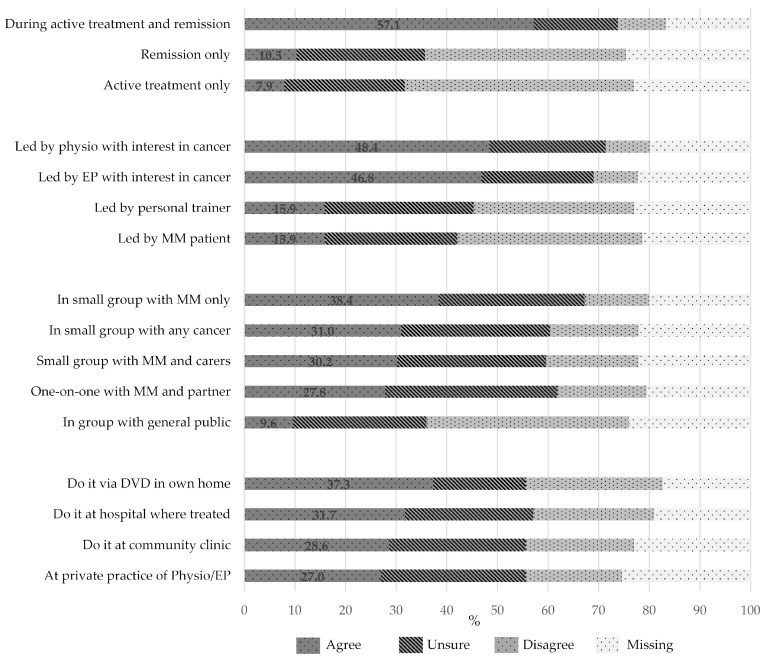
Preferences of respondents for timing, location, and composition of an exercise program (*n* = 126). Physio, physiotherapist; EP, exercise physiologist; MM, multiple myeloma.

Fifty-five percent of respondents were interested in participating in an exercise program. There was no significant difference in interest based on treatment stage (*p* = 0.35). A similar number of respondents expressed a preference to participate in exercise programs during active treatment and in remission (57.1%) (Figure 3). The level of agreement was higher for exercise sessions to be supervised by an exercise professional with a special interest in cancer, compared to personal trainers (15.9%) or peers with cancer (15.9%), with no difference in preference between a physiotherapist (48.4%) and an exercise physiologist (46.8%). There was no clear preference for whether the exercise program included their partner (36.6%) or a social component (31.5%).

There was a very strong preference for an exercise program with flexible times (73.6%), that was low-cost (77.4%), with parking always available (68.8%), and at a location close to home (68.8%). There was no clear preference for the location of the exercise program—i.e., whether at a hospital, community clinic, private practice of exercise professional, or in the patient’s own home with a DVD. Similarly, there was no clear preference for whether the sessions were one-on-one versus in a small group. However, where a small group exercise program was offered, there was a preference for the group to comprise other people with MM or other cancers, rather than with the general public (Figure 3).

## 4. Discussion

This study explored PA participation among people with MM, including differences by treatment stage, MM symptoms, associated demographic factors, and PA advice from healthcare professionals. Interest in, and preferences for, an exercise program were also determined. PA levels significantly reduced after a diagnosis of MM, but were not associated with whether people with MM were on-treatment or in remission, what MM symptoms they were experiencing, or whether a healthcare professional discussed PA with them. Indeed, the only significant predictor for PA after diagnosis was pre-diagnosis PA level. People with MM are interested in participating in exercise programs during active treatment and in remission, which are supervised by exercise specialists with an interest in cancer, and offered one-on-one or in small groups of people with MM or other cancers.

The proportion of respondents reporting meeting PA guidelines was lower following a diagnosis of MM than pre-diagnosis. This may have been speculated to be due to the debilitating side effects of its treatments. There is evidence that people with MM experience more symptoms than people with other haematological cancers, though the older age of the MM cohort may be a contributing factor [15]. In the current study, the majority (84.5%) of people with MM reported experiencing at least one symptom to a moderate intensity, most commonly fatigue, back pain, lethargy, and tiredness due to lack of sleep. However, after Bonferroni correction, experiencing MM symptoms to a moderate or greater intensity was not associated with meeting PA guidelines. PA participation did not appear to be influenced by whether people with MM were on-treatment or in remission. Collectively, this suggests that treatment-related side effects do not appear to be a major contributing factor to the lower proportion of people with MM meeting PA guidelines following a diagnosis of MM.

Concerns regarding the safety of PA and exercise for people with MM may contribute, at least in part, to the lower proportion meeting PA guidelines following a MM diagnosis. Indeed, concerns regarding physical over-exertion and psychological stress for people with cancer have been identified by health care providers as limiting PA discussions [16]. Despite 70% of people with MM presenting with osteoporosis and osteolytic bone lesions at diagnosis [17], current research suggests that supervised exercise is safe and feasible for people with these conditions, including advanced cancer populations [18,19].

Recommendations for PA from the treating haematologist may have been thought to mitigate the perceived risks of participating in PA for people with MM [13]. Coon and Coleman [20] interviewed 21 people with MM and reported that advice from a clinician was an extrinsic facilitator to participation in an exercise program. However, Kenzik et al. found that discussions of PA by physicians were more likely in younger cancer survivors, with survivors aged ≥65 years less likely to report discussing strategies to improve health [21]. Given that most people with MM are over 70 years of age, this may have explained, at least in part, why so few people with MM in the present study reported receiving PA and exercise advice (42.1%), or being referred to an exercise specialist (13.5%).

People with MM reported a high interest in receiving advice on PA and exercise. Despite this, only 5% of respondents reported asking their clinician for a referral to an exercise specialist. Providing more disease-specific education around the benefits of exercise may encourage people with MM to take the initiative to discuss PA with their treating healthcare team. This may be especially important for those who have not exercised in the past, where more education and guidance may be necessary to enhance uptake and ensure continued engagement.

An exercise program that included flexible session times, was low-cost, and located close to home with access to parking was of high interest for people with MM. The high number and severity of MM treatment side effects and disease complications may explain, at least in part, the desire for flexible programs that take into account general health on any particular day. This is consistent with the study by Craike et al. [13], who found that people treated for MM in the past 2–12 months (*n* = 24) reported a strong preference for an individualised and flexible PA program. There was also a strong preference for programs to be guided by a physiotherapist or exercise physiologist with an interest in cancer, perhaps highlighting their fear of injury or lack of self-efficacy due to their disease and treatment complications. Exercise programs were equally appealing whether offered one-on-one or in small groups of people with MM or other cancers. The high proportion of respondents who travelled to their medical consultations using private vehicles (82.5%) is consistent with the desire for access to parking. Collectively, these factors should be considered to optimize feasibility and acceptability when designing an exercise intervention or program for people with MM.

Strengths of this study include the strong person-centred focus and inclusion of a vast range of factors that may influence PA participation in people with MM. The sample accurately represented people with MM in Australia, including people treated at public and private hospitals/clinics, respondents from all education levels, and representation from all disease stages, including during relapsed disease (44%) when the frequency of disease-related symptoms is greatest [22]. Historically, there has been a tendency for younger, fitter individuals to participate in these types of studies. For example, only 21.6% of people with MM in the previous study by Craike et al. [9] were over 70 years. In contrast, 44% of the respondents in the present study were aged over 70 years, suggesting that the typical underestimation of older adults in these types of studies was not apparent.

A limitation of this study is the recruitment of participants via outpatient hospitals/clinics and community seminars, which would have been limited to people with MM who were well enough to attend. Consequently, the views of people with MM who were too unwell to attend outpatient clinics were not captured, possibly excluding more symptomatic and globally less-well individuals. In addition, there was no representation from rural areas. With the recent increased uptake of telemedicine, there may be increased interest in using this technology to reach people with MM in rural locations; so, collecting data on their PA preferences would be beneficial. Furthermore, we relied on retrospective assessment of PA participation before diagnosis, which is vulnerable to social desirability and recall bias. In addition, clinical information, including disease stage, time since diagnosis, and treatments received was self-reported and not validated. The incidence of co-morbidities, which may affect PA participation, was also not collected. Finally, the survey did not assess all possible PA context preferences.

This study has provided crucial information on the factors influencing PA participation following an MM diagnosis, including the association of MM symptoms with current PA. Consideration of the needs and preferences of people with MM for exercise programs may encourage higher rates of participation. A greater understanding of the contributors to the rapid drop in PA participation at diagnosis, and strategies to increase PA and thus increase the quality of life of people with MM, are urgently required.

## Figures and Tables

**Table 1:** Characteristics of the survey respondents (*n* = 126).

	n (%)
Gender (male)	83 (65.9)
Age (years)	
<40	1 (0.8)
40–49	8 (6.3)
50–59	23 (18.3)
60–69	38 (30.2)
70–79	43 (34.1)
≥80	13 (10.3)
Living arrangements	
Partner/spouse	80 (63.5)
Partner/spouse and children	21 (16.7)
Alone	25 (19.8)
Mode of transport to medical appointments ^a^	
Private vehicle	104 (82.5)
Public transport	12 (9.5)
Other (taxi/Uber, community service, ambulance)	24 (19.0)
Highest level of education	
Secondary school to year 10	49 (38.9)
Secondary school to year 12	27 (21.4)
Certificate or Diploma	29 (23.0)
University degree	18 (14.3)
Area of residence	
Metropolitan area	98 (77.8)
Regional/rural area	28 (22.2)
Location of recruitment	
Public clinic	81 (64.3)
Private clinic	32 (25.4)
Other	13 (10.3)
Treatment stage	
Smouldering	6 (4.8)
Newly-diagnosed	33 (26.2)
Non-active	31 (24.6)
Relapsed	56 (44.4)
MM treatment received	
Autologous stem cell transplant	51 (40.5)
Chemotherapy	76 (60.3)
Radiotherapy	22 (17.5)
Bisphosphonates	73 (57.9)
Other (e.g., steroids, Revlimid, Thalidomide)	115 (91.3)

^a^ For transport methods, more than one response was possible. MM, multiple myeloma.

**Table 2:** Logistic regression predicting the likelihood of participating in sufficient physical activity among people with multiple myeloma (*n* = 88).

							95% CI	
	Coeff	SE	df	z	*p*	Odds Ratio	Lower	Upper
Pre-diagnosis physical activity	3.84	1.60	1	2.40	0.02	46.7	2.03	1072.1
Current age	−0.06	0.08	1	−0.70	0.48	0.94	0.80	1.11
Male gender	2.97	1.99	1	1.49	0.14	19.5	0.40	958.0
Time since diagnosis	0.06	0.16	1	0.39	0.69	1.07	0.77	1.47
Disease stage (referent Newly-diagnosed)								
Relapsed	3.27	2.70	1	1.21	0.23	26.3	0.13	5260.4
Non-active	1.78	1.96	1	0.91	0.37	5.93	0.13	279.2
Education level (referent completed Year 10)								
Completed Year 12	0.01	1.74	1	0.00	1.00	1.01	0.03	30.3
Certificate or Diploma	−1.05	2.02	1	−0.52	0.60	0.35	0.01	18.4
University degree	2.13	1.90	1	1.12	0.26	8.38	0.20	345.9
Living situation (referent Living alone)								
Living with partner	−0.45	1.33	1	−0.34	0.73	0.63	0.05	8.65
Living with adult children/family	−1.65	2.41	1	−0.69	0.49	0.19	0.002	21.5
Location of residence (referent Regional)								
Metropolitan	−1.43	1.47	1	−0.97	0.33	0.24	0.01	4.28
Physical activity advice from HCP	−0.92	1.29	1	−0.71	0.48	0.40	0.03	5.01
MM symptoms to at least moderate intensity	−3.33	1.81	1	−1.84	0.07	0.04	0.001	1.25
Referral to exercise professional	3.31	1.88	1	1.77	0.08	27.5	0.70	1085.8
(Constant)	−2.36							

CI, confidence interval; Coeff, coefficient; SE, standard error; df, degrees of freedom; z, z-statistic; *p*, *p* value; HCP, healthcare professional; MM, multiple myeloma.

## Supplementary Materials

The following are available online at https://www.mdpi.com/2077-0383/9/10/3277/s1, Table S1: Survey responses for symptoms experienced by respondents during the previous month; Table S2: Survey responses for respondents for frequency of receiving physical activity and exercise advice from healthcare professionals; Table S3: Survey responses by respondents for frequency of recommendations to see an exercise specialist for physical activity and exercise advice; Table S4: Survey responses by respondents for preferences for an exercise program for people with multiple myeloma; Table S5: Survey responses by respondents for components of an exercise program for people with multiple myeloma.

## References

[1] Ramsenthaler C., Kane P., Gao W., Siegert R.J., Edmonds P.M., Schey S.A., Higginson I.J. (2016). Prevalence of symptoms in patients with multiple myeloma: A systematic review and meta-analysis. Eur. J. Haematol..

[2] Palumbo A., Anderson K. (2011). Multiple myeloma. N. Engl. J. Med..

[3] Snowden J.A., Greenfield D.M., Bird J.M., Boland E., Bowcock S., Fisher A., Low E., Morris M., Yong K., Pratt G. (2017). Guidelines for screening and management of late and long-term consequences of myeloma and its treatment. Br. J. Haematol..

[4] Kent E.E., Ambs A., Mitchell S.A., Clauser S.B., Smith A.W., Hays R.D. (2015). Health-related quality of life in older adult survivors of selected cancers: Data from the SEER-MHOS linkage. Cancer.

[5] Groeneveldt L., Mein G., Garrod R., Jewell A.P., Someren K.V., Stephens R., D’Sa S.P., Yong K.L. (2013). A mixed exercise training programme is feasible and safe and may improve quality of life and muscle strength in multiple myeloma survivors. BMC Cancer.

[6] Jones L.W., Courneya K.S., Vallance J.K., Ladha A.B., Mant M.J., Belch A.R., Stewart D.A., Reiman T. (2004). Association between exercise and quality of life in multiple myeloma cancer survivors. Supportive Care Cancer.

[7] Shallwani S., Dalzell M.-A., Sateren W., O’Brien S. (2015). Exercise compliance among patients with multiple myeloma undergoing chemotherapy: A retrospective study. Supportive Care Cancer.

[8] Servadio M., Cottone F., Sommer K., Oerlemans S., van de Poll-Franse L., Efficace F. (2019). Physical activity and health-related quality of life in multiple myeloma survivors: The PROFILES registry. BMJ Supportive Palliat. Care.

[9] Craike M., Hose K., Livingston P. (2013). Physical activity participation and barriers for people with multiple myeloma. Supportive Care Cancer.

[10] Godin G. (2011). The Godin–Shephard Leisure–Time Physical Activity Questionnaire. Health Fit. J. Can..

[11] Boyle T., Lynch B.M., Courneya K.S., Vallance J.K. (2015). Agreement between accelerometer-assessed and self-reported physical activity and sedentary time in colon cancer survivors. Supportive Care Cancer.

[12] Australian Institute of Health and Welfare 2019 Insufficient Physical Activity. https://www.aihw.gov.au/reports/risk-factors/insufficient-physical-activity.

[13] Craike S.M., Hose J.K., Courneya M.K., Harrison M.S., Livingston M.P. (2017). Physical activity preferences for people living with multiple myeloma: A qualitative study. Cancer Nurs..

[14] Australian Institute of Health and Welfare 2017 Cancer in Australia 2017. https://www.aihw.gov.au/getmedia/3da1f3c2-30f0-4475-8aed-1f19f8e16d48/20066-cancer-2017.pdf.aspx?inline=true.

[15] Johnsen A.T., Tholstrup D., Petersen M.A., Pedersen L., Groenvold M. (2009). Health related quality of life in a nationally representative sample of haematological patients. Eur. J. Haematol..

[16] Haussmann A., Gabrian M., Ungar N., Jooss S., Wiskemann J., Sieverding M., Steindorf K. (2018). What hinders healthcare professionals in promoting physical activity towards cancer patients? The influencing role of healthcare professionals’ concerns, perceived patient characteristics and perceived structural factors. Eur. J. Cancer Care.

[17] Roodman G.D. (2009). Pathogenesis of myeloma bone disease. Leukemia.

[18] Heywood R., McCarthy A.L., Skinner T.L. (2017). Safety and feasibility of exercise interventions in patients with advanced cancer: A systematic review. Supportive Care Cancer.

[19] Galvao D.A., Taaffe D.R., Spry N., Cormie P., Joseph D., Chambers S.K., Chee R., Peddle-McIntyre C.J., Hart N.H., Baumann F.T. (2018). Exercise preserves physical function in prostate cancer patients with bone metastases. Med. Sci. Sports Exerc..

[20] Coon S.K., Coleman E.A. (2004). Keep moving: Patients with myeloma talk about exercise and fatigue. Oncol. Nurs. Forum.

[21] Kenzik K., Pisu M., Fouad M.N., Martin M.Y. (2016). Are long-term cancer survivors and physicians discussing health promotion and healthy behaviors?. J. Cancer Surviv..

[22] Ramsenthaler C., Osborne T.R., Gao W., Siegert R.J., Edmonds P.M., Schey S.A., Higginson I.J. (2016). The impact of disease-related symptoms and palliative care concerns on health-related quality of life in multiple myeloma: A multi-centre study. BMC Cancer.

