# Supplementary material for: Physical Activity in People with Multiple Myeloma: Associated Factors and Exercise Program Preferences
Source: J Clin Med. 2020 Oct 13;9(10):3277. doi: 10.3390/jcm9103277 (PMC7601964; doi:10.3390/jcm9103277)
Supplement: Supplementary file 1 [file jcm-09-03277-s001.zip › jcm-949361-supplementary.docx]

**Supplementary Data – Survey Items and Responses**

**Table 1.** Survey responses for symptoms experienced by respondents during the previous month.

| **Survey Item** | **Not at all** | | **Slightly** | | **Moderately** | | **Severely** | | **Over-** | |
| --- | --- | --- | --- | --- | --- | --- | --- | --- | --- | --- |
|  |  |  |  |  |  |  |  |  | **whelmingly** | |
| **Below is a list of symptoms that you may or may not have experienced. For each symptom, please tick the box that best describes how it has affected you over the past month.** | n | % | n | % | n | % | n | % | n | % |
| Fatigued easily | 20 | 15.9 | 27 | 21.4 | 38 | 30.2 | 29 | 23.0 | 4 | 3.2 |
| Back pain | 28 | 22.2 | 29 | 23.0 | 30 | 23.8 | 25 | 19.8 | 2 | 1.6 |
| Lethargy | 31 | 24.6 | 23 | 18.3 | 38 | 30.2 | 15 | 11.9 | 2 | 1.6 |
| Tiredness due lack of sleep | 31 | 24.6 | 30 | 23.8 | 36 | 28.6 | 17 | 13.5 | 1 | 0.8 |
| Nerve symptoms | 37 | 29.4 | 26 | 20.6 | 36 | 28.6 | 14 | 11.1 | 3 | 2.4 |
| Breathlessness on exertion | 27 | 21.4 | 34 | 27.0 | 33 | 26.2 | 17 | 13.5 | 3 | 2.4 |
| Muscle weakness | 28 | 22.2 | 34 | 27.0 | 31 | 24.6 | 16 | 12.7 | 2 | 1.6 |
| Forgetfulness | 46 | 36.5 | 30 | 23.8 | 29 | 23.0 | 8 | 6.3 | 2 | 1.6 |
| Other bone pain eg. ribs | 45 | 35.7 | 25 | 19.8 | 19 | 15.1 | 16 | 12.7 | 0 | 0.0 |
| Poor balance | 48 | 38.1 | 34 | 27.0 | 24 | 19.0 | 9 | 7.1 | 1 | 0.8 |
| Dizziness | 69 | 54.8 | 24 | 19.0 | 15 | 11.9 | 4 | 3.2 | 0 | 0.0 |
| Breathlessness at rest | 73 | 57.9 | 21 | 16.7 | 12 | 9.5 | 4 | 3.2 | 0 | 0.0 |
| Nausea | 69 | 54.8 | 29 | 23.0 | 9 | 7.1 | 4 | 3.2 | 0 | 0.0 |
|  |  |  |  |  |  |  |  |  |  |  |

**Table 2.** Survey responses for respondents for frequency of receiving physical activity and exercise advice from healthcare professionals.

| Survey Item | No | | Unsure | | Yes | |
| --- | --- | --- | --- | --- | --- | --- |
|  |  |  |  |  |  |  |
|  | n | % | n | % | n | % |
| **Have you received physical activity and exercise advice from a health care professional e.g. your myeloma specialist, rehabilitation specialist, GP, physiotherapist, exercise physiologist, nurse, since your myeloma diagnosis?** | 60 | 47.6 | 8 | 6.3 | 53 | 42.1 |

**Table 3.** Survey responses by respondents for frequency of recommendations to see an exercise specialist for physical activity and exercise advice.

| **Survey Item** | **Never** | | **Rarely** | | **Occasionally** | | **Always** | | **Only when** | |
| --- | --- | --- | --- | --- | --- | --- | --- | --- | --- | --- |
|  |  |  |  |  |  |  |  |  | **I asked** | |
| **Thinking about your consultations specifically with your myeloma specialist, how often have you:** | n | % | n | % | n | % | n | % | n | % |
| Been recommended to see an exercise specialist (e.g. exercise physiologist/physiotherapist) for physical activity and exercise advice? | 74 | 58.7 | 18 | 14.3 | 14 | 11.1 | 3 | 2.4 | 6 | 4.8 |

**Table 4.** Survey responses by respondents for preferences for an exercise programme for people with multiple myeloma.

| **Survey Item** | **Strongly** | | **Disagree** | | **Neither agree** | | **Agree** | | **Strongly** | |
| --- | --- | --- | --- | --- | --- | --- | --- | --- | --- | --- |
|  | **disagree** | |  |  | **nor disagree** | |  |  | **agree** | |
| **If a suitable exercise programme for patients with myeloma was available, what would you prefer? (Please give your opinion for every option)** | n | % | n | % | n | % | n | % | n | % |
| To do it at the hospital where I am treated. | 7 | 5.6 | 23 | 18.3 | 32 | 25.4 | 27 | 21.4 | 13 | 10.3 |
| To do it at a community clinic. | 4 | 3.2 | 23 | 18.3 | 34 | 27.0 | 27 | 21.4 | 9 | 7.1 |
| To do it at the private practice of a physiotherapist or exercise physiologist. | 5 | 4.0 | 19 | 15.1 | 36 | 28.6 | 24 | 19.0 | 10 | 7.9 |
| To do it via instructions on a DVD in my own home. | 12 | 9.5 | 22 | 17.5 | 23 | 18.3 | 38 | 30.2 | 9 | 7.1 |
| It to be led by a physiotherapist with an interest in cancer. | 5 | 4.0 | 6 | 4.8 | 29 | 23.0 | 42 | 33.3 | 19 | 15.1 |
| It to be led by an exercise physiologist with an interest in cancer. | 5 | 4.0 | 6 | 4.8 | 28 | 22.2 | 35 | 27.8 | 24 | 19.0 |
| It to be led by a personal trainer with a certificate to provide general exercise advice. | 10 | 7.9 | 30 | 23.8 | 37 | 29.4 | 15 | 11.9 | 5 | 4.0 |
| It to be led by a patient with myeloma. | 11 | 8.7 | 35 | 27.8 | 33 | 26.2 | 17 | 13.5 | 3 | 2.4 |
| It to be in a group with participants from the general public. | 9 | 7.1 | 41 | 32.5 | 33 | 26.2 | 12 | 9.5 | 0 | 0.0 |
| It to be in a small group (3-10 others) which included only participants with any type of cancer. | 5 | 4.0 | 17 | 13.5 | 37 | 29.4 | 35 | 27.8 | 4 | 3.2 |
| It to be done in a small group which included only participants with myeloma. | 6 | 4.8 | 10 | 7.9 | 36 | 28.6 | 43 | 34.1 | 5 | 4.0 |
| It to be done in a small group that included participants with myeloma and their partners or carers. | 7 | 5.6 | 16 | 12.7 | 37 | 29.4 | 34 | 27.0 | 4 | 3.2 |
| It to be done one-on-one with just you and your partner or carer. | 6 | 4.8 | 16 | 12.7 | 43 | 34.1 | 26 | 20.6 | 9 | 7.1 |
| To do it during both active treatment and remission. | 4 | 3.2 | 8 | 6.3 | 21 | 16.7 | 42 | 33.3 | 30 | 23.8 |
| To do it during active treatment only and not during remission. | 12 | 9.5 | 45 | 35.7 | 30 | 23.8 | 8 | 6.3 | 2 | 1.6 |
| To do it during remission only and not during active treatment. | 14 | 11.1 | 36 | 28.6 | 32 | 25.4 | 11 | 8.7 | 2 | 1.6 |
| Not to do it at all | 42 | 33.3 | 26 | 20.6 | 15 | 11.9 | 10 | 7.9 | 7 | 5.6 |

**Table 5.** Survey responses by respondents for components of an exercise programme for people with multiple myeloma.

| **Survey Item** | **Very** | | **Unimportant** | | **Neither important** | | **Important** | | **Very** | |
| --- | --- | --- | --- | --- | --- | --- | --- | --- | --- | --- |
|  | **unimportant** | |  | | **nor unimportant** | |  |  | **important** | |
| **Please indicate which component/s of an exercise programme for patients with multiple myeloma would be important for you to participate.** | n | % | n | % | n | % | n | % | n | % |
| Parking is always available. | 2 | 1.6 | 7 | 5.6 | 18 | 14.3 | 37 | 29.4 | 49 | 38.9 |
| Parking is free. | 2 | 1.6 | 8 | 6.3 | 22 | 17.5 | 37 | 29.4 | 45 | 35.7 |
| It is close to my home. | 1 | 0.8 | 5 | 4.0 | 22 | 17.5 | 46 | 36.5 | 40 | 31.7 |
| There are flexible times for attendance. | 1 | 0.8 | 3 | 2.4 | 17 | 13.5 | 51 | 40.5 | 41 | 32.5 |
| The programme is offered at low cost. | 1 | 0.8 | 4 | 3.2 | 8 | 6.3 | 50 | 39.7 | 46 | 36.5 |
| My partner or carer can participate as well. | 3 | 2.4 | 21 | 16.7 | 35 | 27.8 | 25 | 19.8 | 20 | 15.9 |
| An optional social programme is included e.g. coffee with other participants after the session. | 3 | 2.4 | 20 | 15.9 | 47 | 37.3 | 29 | 23.0 | 10 | 7.9 |
